# Co-Ultra PEALut Enhances Endogenous Repair Response Following Moderate Traumatic Brain Injury

**DOI:** 10.3390/ijms22168717

**Published:** 2021-08-13

**Authors:** Michela Campolo, Rosalia Crupi, Marika Cordaro, Salvatore Massimo Cardali, Alessio Ardizzone, Giovanna Casili, Sarah Adriana Scuderi, Rosalba Siracusa, Emanuela Esposito, Alfredo Conti, Salvatore Cuzzocrea

**Affiliations:** 1 Department of Chemical, Biological, Pharmaceutical and Environmental Sciences, University of Messina, 98166 Messina, Italy; campolom@unime.it (M.C.); rosalia.crupi@unime.it (R.C.); marika.cordaro@unime.it (M.C.); aleardizzone@unime.it (A.A.); gcasili@unime.it (G.C.); sarahadriana.scuderi@unime.it (S.A.S.); rsiracusa@unime.it (R.S.); eesposito@unime.it (E.E.); 2 Department of Neurosurgery, University of Messina, 98166 Messina, Italy; salvatore.cardali@unime.it; 3 Dipartimento di Scienze Biomediche e Neuromotorie, Alma Mater Studiorum University of Bologna, 40126 Bologna, Italy; alfredo.conti2@unibo.it; 4 IRCCS Istituto delle Scienze Neurologiche di Bologna, 40139 Bologna, Italy; 5 Department of Pharmacological and Physiological Science, Saint Louis University, Saint Louis, MO 63104, USA

**Keywords:** traumatic brain injury, palmitoylethanolamide, luteolin, neurogenesis, mice, patients

## Abstract

This study aimed to assess the neuro-regenerative properties of co-ultramicronized PEALut (Glialia^®^), composed of palmitoylethanolamide (PEA) and the flavonoid luteolin (Lut), in an in vivo model of traumatic brain injury (TBI) and patients affected by moderate TBI. An increase in neurogenesis was seen in the mice at 72 h and 7 d after TBI. The co-ultra PEALut treatment helped the neuronal reconstitution process to restore the basal level of both novel and mature neurons; moreover, it induced a significant upregulation of the neurotrophic factors, which ultimately led to progress in terms of memory recall during behavioral testing. Moreover, our preliminary findings in a clinical trial suggested that Glialia^®^ treatment facilitated neural recovery on working memory. Thus, co-ultra PEALut (Glialia^®^) could represent a valuable therapeutic agent for intensifying the endogenous repair response in order to better treat TBI.

## 1. Introduction

Traumatic Brain Injury (TBI) is a leading cause of disability and death in young adults of industrialized countries [1,2]. Brain trauma is due to the application of an external physical force, which causes functional impairment of a temporary or permanent type [3].

During the weeks following a trauma, the central nervous system (CNS) maintains its original capacity for regeneration, carrying out secondary compensatory mechanisms designed to help with the lesion [4]. In this context, one possible mechanism is characterized by the neurogenesis process [5,6,7,8,9], which acts in a central role in the formation of new nerve cells from neural stem cells or progenitor cells [10].

The correlation between TBI and neurogenesis processes has been extensively investigated in the last decade [11,12].

This link could constitute a new key for a more complete understanding of the molecular mechanisms underlying brain damage, thus leading to the development of new pharmacological strategies.

In this regard, it has been demonstrated how the regulation of brain tissue repair processes could promote neuronal recovery following brain insults [13], contributing to the re-establishment of physiological homeostasis in the CNS [14].

On these scientific bases, the present study aimed to consider the neuroregenerative properties of a compound comprised of palmitoylethanolamide (PEA), an endogenous fatty acid amide member of the N-acylethanolamines family, and the vegetable flavonoid luteolin (Lut), known as co-ultramicronized PEALut (co-ultra PEALut). 

PEA is abundant in the CNS and it represents a potent neuroprotective agent [15,16,17]; it has been shown that PEA re-established the behavioral phenotype and normalized the biochemical and functional changes occurring in traumatic injury [18].

Relatedly, PEA would have the ability to act on the synaptic plasticity of the hippocampus and neurogenesis thanks to the interaction with the monoamine system [19], modulating neurotrophic factors levels [20], and neural cell death [21] as well as gliosis [22]. 

On other hand, also Lut has several pharmacological actions, including antioxidant properties, a memory-improving effect [23,24,25] and neurotrophic potential, which are extremely beneficial in the field of neurodegenerative disorders [25,26] and a broad-spectrum of CNS diseases.

Lut, alone or in combination, has proven to be useful in the treatment of various CNS traumatic injuries [27], including TBI [28,29]. Lut exerts neuroprotective effects through the modulation of different signaling pathways, influencing neurons’ survival [29], increasing BDNF expression [30] and promoting antioxidant response against intracellular reactive oxygen species (ROS) in neurons [29,31].

Interestingly, although PEA has no antioxidant effects per se, its co-ultramicronization with Lut results in a compound that demonstrates the best properties of both original molecules. 

Previously, the association between these two molecules, in a fixed dose of 10:1 in mass, has revealed potent neuroprotective activity in neurodegenerative diseases [32,33,34], as well as in traumatic CNS damage [35,36]. Based on these findings, we hypothesized that co-ultra PEALut would be an effective treatment in TBI, maybe modulating neurogenesis following TBI. 

The study was conducted first in a CCI mouse model to evaluate neurogenesis parameters; then, in the second part, the study was designed to evaluate the effects, in terms of functional recovery, of co-ultra PEALut in patients with moderate TBI. 

## 2. Results

### 2.1. Pre-Clinical Results

#### 2.1.1. Co-Ultra PEALut Reduced Neuronal Degeneration in the Hippocampus and Neurological Deficit

**Figure 1:**
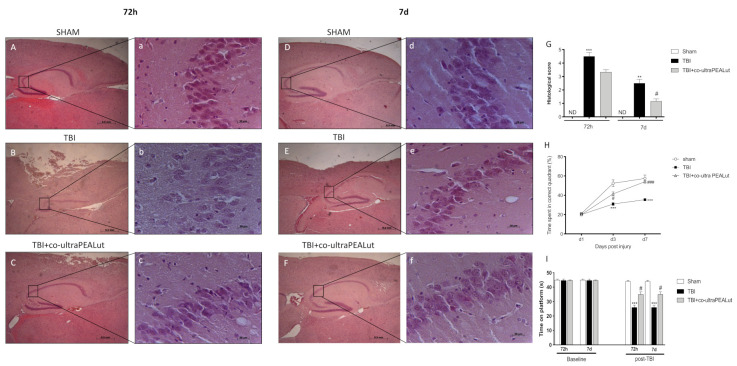
Effect of co-ultra PEALut on hippocampal morphology and spatial learning 72 h and 7 d after TBI. As compared with sham groups at 72 h and 7 d (**A**,**D**, respectively, see histological score **G**), TBI induced a significant alteration of tissue morphology (**B**,**E**, respectively, see histological score **G**) in the DG and CA3 region. Co-ultra PEALut treatment restored markedly hippocampal tissue. Scale bar = 0.5 mm (**A**–**F**) and 20 μm (**a**–**f**) for higher magnification. One-way ANOVA test. # *p* < 0.05 vs. corresponding TBI. ** *p* < 0.01 vs. corresponding sham group. *** *p* < 0.001 vs. corresponding sham group (F_72h_ = 147.3 and F_7d_ = 42.25). TBI significantly impaired spatial learning at 72 h and 7 d compared to sham controls. Co-ultra PEALut improves spatial learning performance (**H**). *** *p* < 0.05 vs. Saline group. # *p* < 0.05 vs. TBI (F = 067). At 72 h and 7 d after TBI, animals showed significant impairments in motor deficits assayed by rotarod test. Co-ultra PEALut post-TBI significantly improved motor function evaluated by the rotarod task (**I**). One-way (**G**,**I**) and Two-way (H) ANOVA test. Data are expressed as Mean ± SEM from N = 10 mice for each group. *** *p* < 0.001 vs. Sham group; # *p* < 0.05 vs. TBI; ### *p* < 0.001 vs. TBI.

Sections were stained with H&E to observe the longitudinal brain level comprising the dorsal hippocampus (subjacent to contusion). Brain sections from the TBI groups at 72 h revealed not only a flattening of the hippocampus but showed a large number of degenerating neurons in the pyramidal layers (particularly in the CA3 area). These cells exhibited stained eosinophilic cytoplasm and pyknotic to fragmented nuclei (Figure 1B, see particle Figure 1b). Neuronal degeneration in TBI groups was noted apparently more at 72 h instead of 7 d (Figure 1E, see particle Figure 1e). No evidence of neuronal degeneration was observed in the H&E sections of sham groups in both 72 h and 7 d (Figure 1A, see particle Figure 1a,D, see particle Figure 1d). Treatment with co-ultra PEALut modulated neuronal degeneration 72 h after TBI (Figure 1C, see particle Figure 1c). The same situation was found in the CA3 area at 7 d where, following co-ultra PEALut treatment, cell differentiation significantly declined compared to the TBI group (Figure 1F, see particle Figure 1f). Histological score is shown in Figure 1G.

Spatial learning was measured by the MWM test that illustrates the mean latency to find the goal platform in the water maze. It was executed respectively during the days post-injury until the sacrifice for both time points (3 and 7 d). The spatial learning function in the TBI mice was notably compromised at both 3 and 7 d after TBI showing a significantly shorter goal latency than compared to sham groups. TBI mice treated with co-ultra PEALut exhibited significant progress in spatial learning at 7 d compared to TBI mice (Figure 1H). To investigate the relationship between neurological deficits in the setting of TBI, we used the rotarod test (considered the most sensitive vestibulomotor test) to evaluate motor function. At 72 h after TBI, mice displayed a range of impairments in locomotor tasks that increased at 7 d. We demonstrated that co-ultra PEALut (1 mg/kg) treatment appreciably ameliorated latency compared to the TBI group both at 72 h and 7 d (Figure 1I).

#### 2.1.2. Co-Ultra PEALut Appreciably Promotes Cell Proliferation

**Figure 2:**
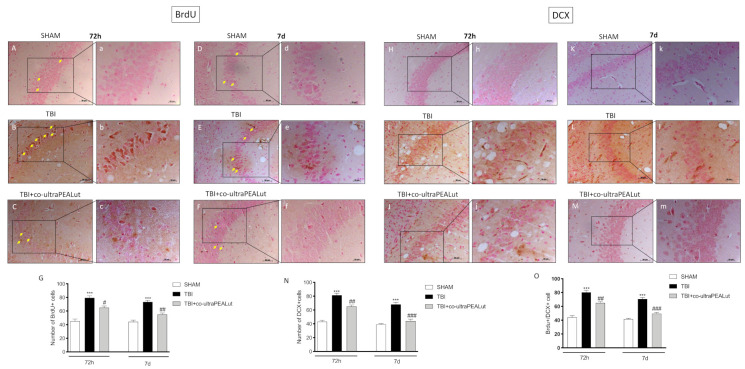
Effect of co-ultra PEALut on newly born cell and mature cell proliferation. Cell proliferation was increased at 72 h and 7 d post TBI (**B**,**E**, respectively, **G**) compared to sham groups (**A**,**D**, respectively, **G**). Co-ultra PEALut treatment modulated this process returning to baseline (**C**,**F**, respectively, **G**). Scale bar = 0.5 mm (**A**–**F**) and 20 μm (**a**–**f**) for higher magnification. One-way ANOVA test. # *p* < 0.05 vs. corresponding TBI group. ## *p* < 0.01 vs. corresponding TBI group. *** *p* < 0.001 vs. corresponding Sham group (F_72h_ = 36.4 and FF_7d_ = 38.7). DCX+ cells increased at 72 h and 7 d post-TBI (**I**,**L**, respectively, **N**) compared to sham groups (**H**,**K** respectively, **N**). Co-ultra PEALut treatment modulated cell maturation comparably to the sham group (**J**,**M** respectively, **N**). BrdU/DCX expressing cell ratio (**O**). Yellow arrows indicate the positive staining for BrdU. For IHC analyses, the reaction was revealed by a chromogenic substrate (DAB), and counterstaining with Nuclear Fast Red. For a graphic display of the densitometric analyses, the percent of positive staining (DAB brown staining) was measured by computer-assisted color image analysis. The percentage area of immunoreactivity (determined by the number of positive pixels) was expressed as percent of the total tissue area (red staining) within five random fields at a 40× magnification. Data are expressed as Mean ± SEM from N = 10 mice for each group. Scale bar = 0.5 mm (**H**–**M**) and 20 μm (**h**–**m**) for higher magnification. One-way ANOVA test. (**N**) ## *p* < 0.01 vs. corresponding TBI. ### *p* < 0.001 vs. corresponding TBI. *** *p* < 0.001 vs. corresponding sham group (F_72h_ = 60.9 and FF_7d_ = 37.9); (**O**) ## *p* < 0.01 vs. corresponding TBI. ### *p* < 0.001 vs. corresponding TBI. *** *p* < 0.001 vs. corresponding Sham group (F_72h_ = 5.57 and FF_7d_ = 73.4).

To determine the response of neural progenitor cells following a moderate TBI insult in the hippocampus, we examined cells undergoing proliferation through BrdU (thymidine analog marker for splitting cells that are integrated into newly replicated DNA during the cell cycle in the S phase) staining. At 72 h post-TBI, the quantity of BrdU positive cells was significantly increased compared to the sham group (Figure 2B,b and Figure 2A,a respectively). The treatment with co-ultra PEALut modulated regeneration process both at 72 h (Figure 2C,c) and 7 d (Figure 2E,e), normalizing neurogenesis (Figure 2F,f), as shown by the number of BrdU almost comparable to control levels. Cell count analysis is shown in Figure 2G.

To label neurons in regeneration, we used DCX antibody. After 3 d of TBI were revealed a marked number of immature cells in the CA3 zone of the hippocampus (Figure 2I,i). Treatment with co-ultra PEALut meaningfully reduced the number of DCX positive cells bringing the cells number comparable to the sham group (Figure 2J,j). Moreover, at 7 d after TBI, was found a spontaneous regeneration of immature cells (Figure 2L,l); Co-ultra PEALut treatment restored the number of DCX positive cells enhancing the process (Figure 2M,m). Cell count analysis is shown in Figure 2N.

The reduction of newborn cells, meaning an acceleration of neurogenesis, was confirmed by the BrdU+/DCX expressing cell ratio which increased in TBI mice and decreased following co-ultra-PEALut administration, which returns the number of newborn cells to the control values (Figure 2O).

#### 2.1.3. The Effects of Co-Ultra PEALut Treatment to Prevent Neurodegeneration

**Figure 3:**
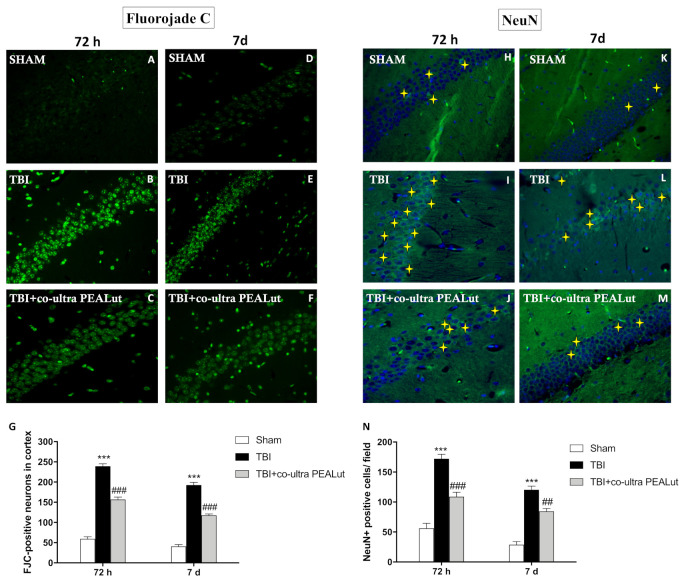
Effect of co-ultra PEALut on neuronal degeneration. Fluoro-Jade-positive neurons were significantly recognized in TBI-72 h (**B**,**G**) and TBI-7 d (**E**,**G**) groups, compared to respective control mice (**A**,**D**,**G**). Co-ultra PEALut reduced the neurodegeneration process at 72 h (**C**,**G**) and 7 d (**F**,**G**), following TBI. A considerable number of NeuN-positive cells were detected in TBI-72 h (**I**,**N**) and TBI-7 d (**L**,**N**) groups, compared to sham groups (**H**,**K**,**N**). Co-ultra PEALut administration considerably reduced NeuN-positive cells after 72 h (**J**,**N**) and 7 d post-TBI (**M**,**N**). Magnification 40×. Yellow stars indicate the positive staining for NeuN. Data are expressed as Mean ± SEM from N = 10 mice for each group. One-way ANOVA test. *** *p* < 0.001 vs. corresponding Sham group; ## *p* < 0.01 vs. corresponding TBI. ### *p* < 0.001 vs. corresponding TBI.

The neurological injury that occurs after a traumatic insult results in cognitive and memory dysfunction, related to neuronal damage occurring in the hippocampus [37]; Fluoro-Jade is a fluorescein-derived fluorochrome that specifically binds to damaged neurons, allowing the detection and quantification of neurodegeneration [38], representing a reliable marker to test neuroprotective agents following TBI [39]. To determine the response of neural progenitor cells following a TBI insult in the hippocampus, we examined the degenerating neuronal cell body marked by Fluoro-Jade. In patches of pyramidal neurons in CA1, CA2 and CA3 of the hippocampus, at 72 h post injury, numerous Fluoro-Jade-positive neurons were identified (Figure 3B,G), compared to sham group (Figure 3A,G). The intensity of stained neurons diminished by 7 d post-injury when only a few stained neurons in the pyramidal layer were detectable (Figure 3E,G). The treatment with co-ultra PEALut reduced the neurodegeneration process at both 72 h (Figure 3C,G) and 7 d (Figure 3F,G) following TBI. 

Neuronal loss is associated with neuronal injury after TBI; thus we evaluated NeuN, a neuronal-specific nuclear protein [40], through immunofluorescence. Interestingly, it has been shown that the total number of neuronal proliferating cells was significantly increased in the hippocampal area from the TBI injury group compared to control mice, and this is explained by the fact that most of the new neuronal cells will become astrocytes and activated microglia [41].

In our study, we confirmed an increase in the number of NeuN-positive cells in the TBI-damaged group, both at 72 h (Figure 3I,N) and 7 d (Figure 3L,N) compared to the control group in the hippocampus area (Figure 3H,K,N). Interestingly, the protective role of co-ultra PEALut treatment emerged in a reduction of NeuN positive cells number, bringing the cells number close to sham group at 72 h after injury (Figure 3J,N); this effect was observed also 7 d after TBI (Figure 3M,N).

#### 2.1.4. Co-Ultra PEALut Treatment Counteracts Neurodegeneration, Slowing down Programmed Cell Death

Neurodegeneration induces upregulation of Beclin 1, a Bcl-2 interacting protein that promotes the autophagy process [42]. Autophagy was renowned in several neurodegenerative diseases and under trauma conditions, leading to the activation of type II programmed cell death [43]. Caspase-3, onto which there is a convergence of the intrinsic and extrinsic apoptotic pathways, is the main executioner of apoptosis [44].

Using Beclin 1 and Caspase-3 as markers to highlight the autophagic promotion of apoptosis in TBI, we co-merged Beclin 1/Caspase-3 positive staining following trauma, at the hippocampal level, through immunofluorescence analysis.

**Figure 4:**
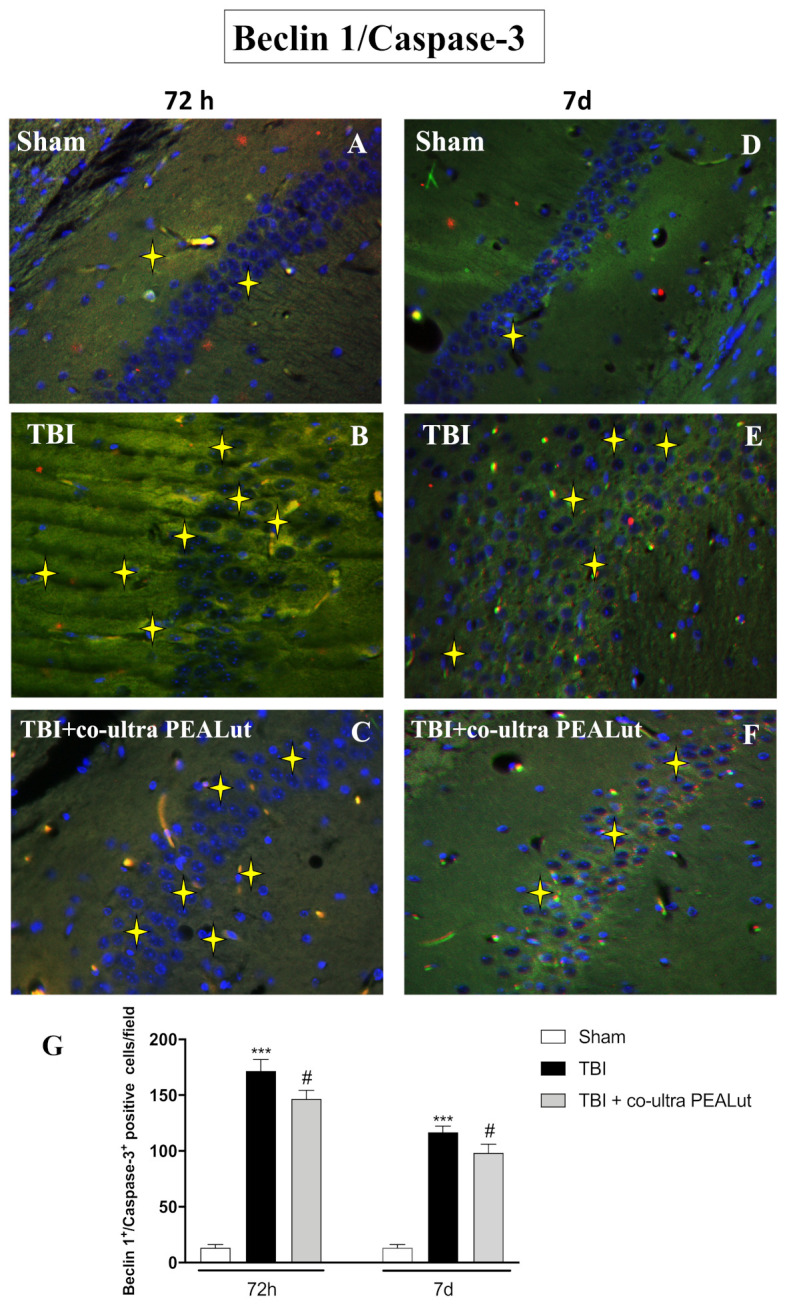
Co-ultra PEALut treatment counteracts neurodegeneration, slowing down programmed cell death. A considerable number of Beclin 1/Caspase-3-positive cells were identified in TBI-72 h (**B**,**G**) and TBI-7 d (**E**,**G**) mice, matched to the corresponding control group (**A**,**D**,**G**). Treatment with co-ultra PEALut reduced this positive expression both 72 h (**C**,**G**) and 7 d (**F**,**G**) post-TBI. Magnification 40×. Yellow stars indicate the positive staining for Beclin 1/Caspase-3. Data are expressed as Mean ± SEM from N = 10 mice for each group. One-way ANOVA test. *** *p* < 0.001 vs. corresponding Sham group; # *p* < 0.05 vs. corresponding TBI.

We demonstrated an increasing number of Beclin 1/Caspase-3 positive-cells near the injury site (Figure 4B,G) compared to the control group (Figure 4A,G); interestingly, Beclin 1/Caspase-3 increasing starts at early stages post-injury (72 h), shrinking slightly at 7 d after trauma (Figure 4E,G). Treatment with co-ultra PEALut significantly reduced the number of Beclin 1/Caspase-3 positive hippocampal neurons at 72 h after injury (Figure 4C,G), with a particular decrease observed 7 d after TBI (Figure 4F,G). These results suggest that cells overexpressing Beclin 1 may exhibit damaged DNA but not yet be directed towards neuronal death; furthermore, the reduction of Beclin 1/Caspase-3 positive staining in co-ultra PEALut group may represent a reduction in autophagy and apoptosis, as a mechanism to discard neurons only partially injured to whole damaged cells, recycling cellular component and reducing brain damage.

#### 2.1.5. Co-Ultra PEALut Administration Reduced Astrogliosis

**Figure 5:**
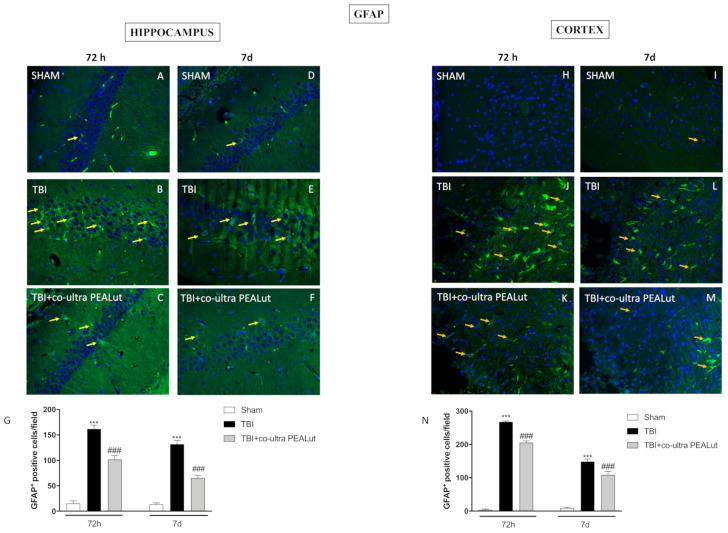
Effect of co-ultra PEALut on astroglial cell activation. GFAP^+^ cells significantly increased at 72 h and 7 d post-TBI in both hippocampus (**B**,**E**,**G**) and cortex (**J**,**L**,**N**); while co-ultra PEALut treatment decreased GFAP+ cells in the hippocampus (**C**,**F**,**G**) and cortex (**K**,**M**,**N**). Sham did not show any astrogliosis (**A**,**D**,**G** and **H**,**I**,**N**). Magnification 40×. Yellow arrows indicate the positive staining for GFAP. Data are expressed as Mean ± SEM from N = 10 mice for each group. One-way ANOVA test. *** *p* < 0.001 vs. corresponding Sham group; ### *p* < 0.001 vs. corresponding TBI.

To analyze the activation of astroglial cells, brain was stained with GFAP antibody. Hippocampal slices of the CA3 area revealed augmented astrogliosis (GFAP^+^ cells) in the TBI group at 72 h and 7 d (Figure 5B,E, respectively) compared to the control group (Figure 5,D respectively). Instead, co-ultra PEALut treatment notably reduced the number of GFAP^+^ cells after 72 h (Figure 5C) that was reported almost to control level at 7 d of TBI (Figure 5F). Densitometric analysis is shown in Figure 5G. 

GFAP expression was also evaluated on the cortex impact area of TBI animals. 72 h and 7 d after the induction of TBI, mice of the vehicle group showed an increased number of GFAP-positive cells (Figure 5J,L), compared to the respective sham groups (Figure 5H,I).

The administration of co-ultra PEALut significantly reduced astrogliosis (Figure 5K,M) both 72 h and 7 d post-TBI. Densitometric analysis is shown in Figure 5N.

#### 2.1.6. Co-Ultra PEALut Administration Reduced Microgliosis

Iba1 immunofluorescence staining was performed in order to evaluate the activation of microglia. 

**Figure 6:**
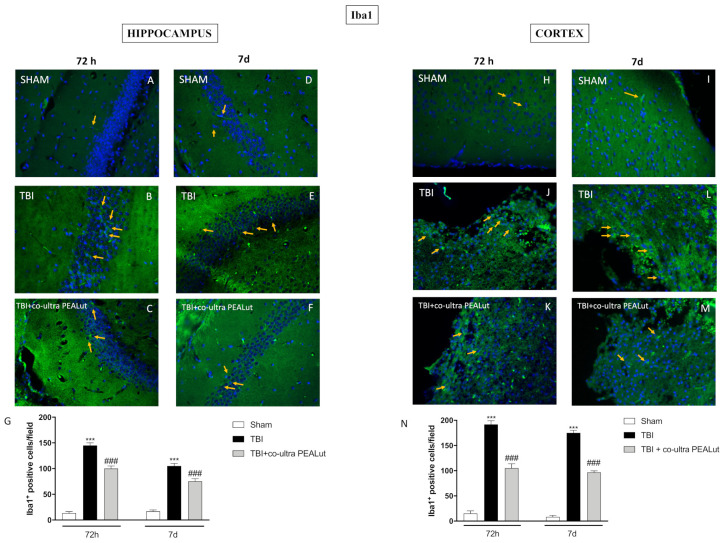
Co-ultra PEALut administration reduced microgliosis. Iba1^+^ cells were significantly increased in the hippocampus (**B**,**E**,**G**) and cortex (**J**,**L**,**N**) of TBI mice, compared to respective sham groups (**A**,**D**,**G** and **H**,**I**,**N**). Co**-**ultra PEALut treatment reduced Iba1^+^ cells expression, at both 72 h and 7 d, in the hippocampus (**C**,**F**,**G**) and cortex (**K**,**M**,**N**). Magnification 40×. Yellow arrows indicate the positive staining for GFAP. Data are expressed as Mean ± SEM from N = 10 mice for each group. One-way ANOVA test. *** *p* < 0.001 vs. corresponding Sham group; ### *p* < 0.001 vs. corresponding TBI.

After the induction of TBI, animals showed an increased expression of Iba1 in the hippocampus (Figure 6B,E) and cortex (Figure 6J,L); on the contrary, this expression was minimal in mice of sham groups (Figure 6A,D,H,I). The administration of co-ultra PEALut has significantly reduced the microgliosis caused by TBI at 72 h and 7 d, in both hippocampus (Figure 6C,F) and cortex (Figure 6K,M) sections. Densitometric analyses are shown in Figure 6G,N.

#### 2.1.7. Co-Ultra PEALut Restored BDNF Basal Level

**Figure 7:**
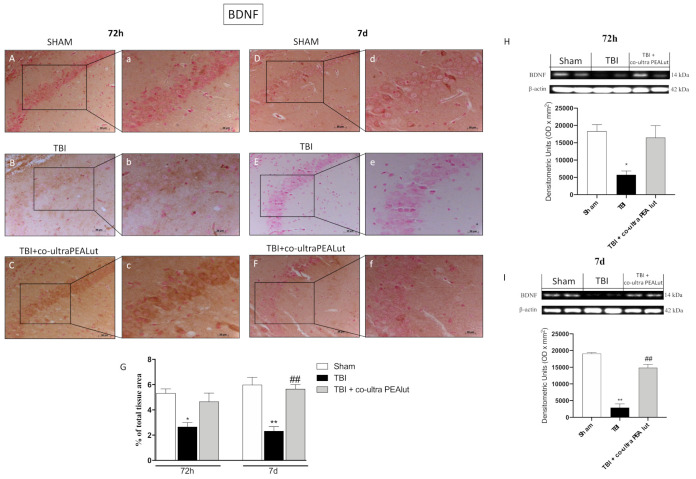
Effect of co-ultra PEALut on BDNF expression. Co-ultra PEALut treatment showed positive staining of BDNF (**C**,**F**,**G**) comparable to sham (**A**,**D**,**G**). After 72 h post-TBI and, more significantly, after 7 d post-TBI, mice showed a reduction in positive BDNF staining (**B**,**E**, respectively, **G**). Data were confirmed by Western blot analysis (**H**,**I**). For IHC analyses, the reaction was revealed by a chromogenic substrate (DAB), and counterstaining with Nuclear Fast Red. For a graphic display of the densitometric analyses, the percent of positive staining (DAB brown staining) was measured by computer-assisted color image analysis. The percentage area of immunoreactivity (determined by the number of positive pixels) was expressed as percent of total tissue area (red staining) within five random fields at a 40× magnification. Scale bar = 0.5 mm (**A**–**F**) and 20 μm (**a**–**f**) for higher magnification (F_72h_ = 8.66 and F_7d_ = 22.2). Data are expressed as Mean ± SEM from N = 20 mice for each group. One-way ANOVA test. * *p* < 0.05 vs. respective Sham; ** *p* <0.01 vs. respective Sham; ## *p* < 0.01 vs. respective TBI.

To examine whether co-ultra PEALut modulates neuronal regeneration through regulation of neurotrophic factors, we have examined BDNF and NT-3 levels in the CA3 zone of the hippocampus by immunohistochemistry (Figure 7). At 72 h and 7 d post-trauma, BDNF-positive staining was significantly reduced (Figure 7B,b and Figure 7E,e respectively) in comparison with the sham group (Figure 7A,a and Figure 7D,d respectively). Surprisingly, co-ultra PEALut, 72 h after trauma, re-established BDNF positive staining almost comparable to sham groups even if 7 d later it is able to restore neurotrophic factors to basal level (Figure 7C,c and Figure 7F,f respectively). Densitometric analysis is shown in Figure 7G. The results obtained from immunohistochemical staining were further confirmed by Western blot analysis (Figure 7H,I).

#### 2.1.8. Co-Ultra PEALut Restored NT-3 Basal Level

**Figure 8:**
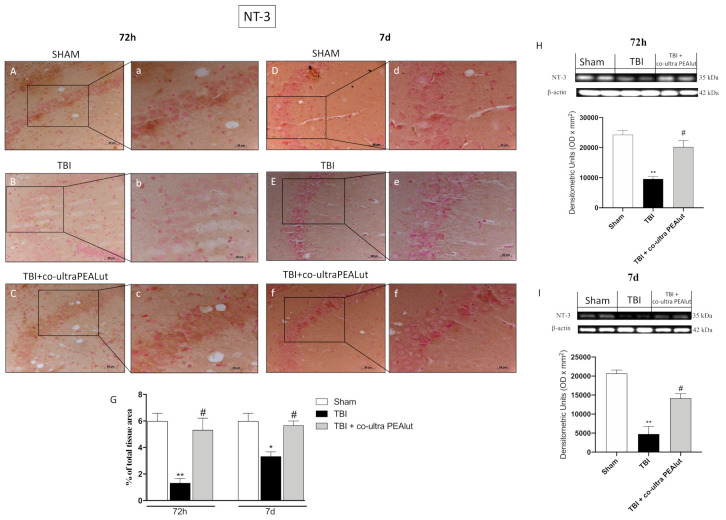
Effect of co-ultra PEALut on NT-3 expression. Co-ultra PEALut administration increased positive staining for NT-3 in a manner comparable to sham 72 h after TBI (**C**,**G**) and much more at 7 d (**F**,**G**). TBI mice showed a reduction in positive NT-3 staining for both 72 h and 7 d (**B**,**E**, respectively, **G**) compared to sham mice (**A**,**D**,**G**). Data were confirmed by Western blot analysis (**H**,**I**). For IHC analyses, the reaction was revealed by a chromogenic substrate (DAB), and counterstaining with Nuclear Fast Red. For a graphic display of the densitometric analyses, the percent of positive staining (DAB brown staining) was measured by computer-assisted color image analysis. The percentage area of immunoreactivity (determined by the number of positive pixels) was expressed as percent of total tissue area (red staining) within five random fields at a 40× magnification. Data are expressed as Mean ± SEM from N = 20 mice for each group. One-way ANOVA test. * *p* < 0.05 vs. respective Sham; ** *p* < 0.01 vs. respective Sham; # *p* < 0.05 vs. respective TBI. Scale bar = 0.5 mm (**H**–**M**) and 20 μm (**h**–**m**) for higher magnification (F_72h_ = 15.64 and F_7d_ = 11.4).

NT-3 showed the same modulation of BDNF. In fact, TBI-72 h and TBI-7 d mice showed a reduced expression of NT-3 (Figure 8B,b and Figure 8E,e, respectively) if compared to corresponding sham groups (Figure 8A,a and Figure 8D,d respectively). On the other hand, co-ultra PEALut, 72 h and 7 d after trauma, significantly preserved sham levels, blocking the reduction of neurotrophic factor NT-3 (Figure 8C,c and Figure 8F,f respectively). Densitometric analysis is shown in Figure 7G. The results obtained from immunohistochemical staining were further confirmed by Western blot analysis (Figure 8H,I).

### 2.2. Clinical Study Results

#### 2.2.1. Basal Characteristics, Treatment and Neurological Assessment of TBI Patients

In the considered period spanning 18 months, 47 patients affected by severe or moderate TBI were admitted and screened for the study purpose (Figure 5). Fourteen (29.8%) were not recruited because they did not meet the inclusion criteria. Three initially enrolled patients were dropped out because of an evolving severe neurological status (Glasgow Outcome Scale 2) and for the poor application or compliance to the study protocol. Basal characteristics of patients are reassumed in Table 1. Finally, 30 patients completed the study in the recruitment period. Accordingly, the power of this superiority study, considering an alpha = 0.05 and a SD of 5 points for the MMSE [45], was equal to 0.30.

Average age was 52 ± 17.5 years across all the patients in the study. The mean age of patients in the study group (standard treatment + Glialia^®^ and control group (standard treatment) were, respectively, 49.7 ± 17.9 years and 53.8 ± 17.5 years (*p* > 0.05).

The initial GCS for the overall patients was 11 ± 1.4; at the last control, GCS was 15 for all patients (*p* < 0.0001). Basal values of GCS in the two groups were similar in the two groups: 11.2 ± 1.3 in the group assuming Glialia^®^ and 11.2 ± 1.4 in the sham group (*p* > 0.05). At the final follow-up, all patients had a GCS of 15. Classifications of CT scan according to the Marshal scale are described in Table 1. Four patients of the study group and 3 of the control group underwent surgical treatment (*p* > 0.05); the surgical procedure consisted of surgical clot evacuation and brain debridement followed by ICP monitoring.

#### 2.2.2. Cognitive Outcome

Cognitive function evaluation performed by MMSE and BNCE showed a significant improvement (*p* < 0.0001) compared to baseline performance. The initial MMSE score in the study group and control group was, respectively, 21.1 ± 4.6 and 21.5 ± 3.1 (*p* > 0.05). After 180 d of follow-up, the analysis of the MMSE scores showed a substantial difference in the improvement in the two groups, with a score of 28.1 ± 2.4 in the study group and 26.1 ± 2.8 in the control patients, with a difference that was statistically significant among the two groups (*p* = 0.04). The improvement of the MMSE score in the group assuming Glialia^®^ was 7.4 ± 1.7, whereas it was 4.6 ± 3.9 for the control group (*p* = 0.02). The patients who achieved a score >24 (normal score) were 15/15 in the study group and 9/15 in the control group with a significantly (*p* = 0.02) larger representation in the study group. The cognitive outcome was analyzed also by the BNCE, assessing different cognitive abilities including working memory, orientation, mental control, incidental recall, clock drawing, and verbal fluency (Table 2 summarized cut off values adjusted for age and education level). Initial BNCE score in the study group and control group was similar (Table 3). It did not reach a statistical difference; however, the test for interference memory showed a better result in patients receiving Glialia^®^ as compared to the control group (*p* = 0.03). Beck’s inventory depression scale score of the last time point was 5.3 ± 3.7 in the study group and 9.9 ± 6.7 in the control patients showing no statistical difference.

#### 2.2.3. Independency Outcome

Patients’ independence and mobility in quotidian living activities (Barthel Index) showed a significant amelioration (*p* < 0.0001) after 180 d of treatment (mean score of 33 ± 8.2 and 98 ± 4.9 at admission and after 180 d, respectively). Barthel indexes, at last follow-up, averaged 98.7 ± 3.6 in the study group and 97 ± 5.9 in the control group. In both groups, the neurological and independence recovery was satisfactory in accordance with standards for moderate TBI patients.

#### 2.2.4. Tolerability

Tolerability to Glialia^®^ treatment was outstanding, with no side events all over the time of the study. Further, regular blood chemistry and hematology tests did not show any deviancies from their standard ranges in relation to Glialia^®^ treatment.

## 3. Discussion

Acute head trauma is followed by increasing cell proliferation and neural differentiation [46]; unfortunately, this spontaneous neurogenesis is not satisfactory to stimulate a significant recovery [47]. This raises the possibility of developing therapeutic strategies aiming at harnessing this neurogenic capacity to repair the damaged brain. Experimental successes in cell transplantation in models of Parkinson’s disease and other neurodegenerative diseases have encouraged TBI researchers to investigate this approach for treating the injured brain providing trophic support to the host tissue to facilitate survival [47].

Many scientific articles [32,35] exposed the neuroprotective abilities of PEA and Lut to improve CNS damage following injuries. On these considerations, in the present study, we investigated the effects of a co-ultra PEALut, consisting of PEA and Lut in a 10:1 ratio, on the neurogenesis features following TBI.

After a brain insult, cell proliferation rises in the DG that increases from three to four times starting two days post-injury [48]. It has been shown that a decreased survival of immature neurons can be seen as early as seven days, while at four weeks post-TBI, both cell proliferation and neuronal differentiation significantly lessened compared to sham mice [49].

Our pre-clinical results suggested that treatment with co-ultra PEALut modulated neurogenesis processes, normalizing the regenerative activity after TBI; moreover, it was able to accelerate the proliferation of NSCs occurred from three days until seven days after trauma, showing a significant restoration of brain tissue and improving spatial learning. 

Several studies showed that PEA alone could have a key part in preserving cellular homeostasis when faced with external stressors like inflammation. Moreover, PEA is efficient in mast cell-mediated models of neuropathic pain and showed its neuroprotective in models of stroke, SCI, TBI, and PD [50,51,52].

Our data demonstrated that three-day post-traumatic treatment with co-ultra PEALut hippocampus damage and cell loss significantly reduced, stimulating neurogenesis. Surprisingly, seven days after trauma, co-ultra PEALut restored hippocampus morphology and promoted neuronal renewal. 

This neuroprotection may contribute to functional recovery following TBI; in fact, we demonstrated that co-ultra PEALut following TBI markedly improved, from day three to seven, the behavioral outcome, revealed in the progress of spatial learning and sensorimotor functional recovery.

These preliminary results confirmed the ability of co-ultraPEALut to modulate the mechanisms underlying neuronal regulation. In particular, these neuromodulatory properties could be due to the interaction of PEA with specific receptor-like Peroxisome Proliferator-Activated Receptors (PPAR)-α receptor, whose function in hippocampal neurogenesis has already been elucidated in the context of brain injury and also closely related to cognitive function [53] as well as its interaction with PEA [54]. In addition, Lut’s antioxidant functions would modulate ROS overload, thus representing a novel way to regulate the increase in oxidative stress related to brain diseases and impaired neurogenesis [55].

Basically, neurogenesis consists of distinctive developmental processes including survival, proliferation and differentiation [56]. Our study displayed that TBI-induced cell proliferation peaks three days after trauma in the DG with a gradual reduction one week later. 

Co-ultra PEALut treatment re-established the number of neurons in the DG following CCI comparable to sham mice, suggesting that co-ultra PEALut could decrease cell proliferation at 72 h or 7 d. 

Relatively, an excessive increase in post-TBI DCX^+^ cells has been associated with epileptogenesis and resulted in a long-term decline in neurogenic capacity [57]. Therefore, targeted suppression of the early post-injury increase in neurogenesis could improve long-term outcomes after brain injury.

After three days of brain trauma, newborn DCX positive cells emigrated toward cerebral cortex lesion [58]; however, co-ultra PEALut treatment clearly modulated DCX positive cells in the DG region. 

The mechanisms underlying this observation might include more optimal preparation of the damaged tissue bed for neuronal regeneration, accelerated removal of debris and toxic products, and/or the enhanced neuronal and dendritic branches maturation. TBI severity affects neurogenesis, increasing neuronal stem cells, immature neuron number and mature neuron generation [59]; the cell proliferation promoted by TBI is aimed at an increase in future astrocytes and activated microglia. This point, in particular, is very important to better explain the neuroprotective action of PEALut treatment that reduced NeuN positive cells [41]. Indeed, co-ultra PEALut has been shown to limit excessive neurogenesis, thus potentially restoring the physiological neuroproliferative capacity. 

Moreover, co-ultra PEALut administration had the ability to reduce neurodegeneration that occurs in the hippocampus following TBI. To further validate the effects of co-ultra PEALut on the degree of neurodegeneration, we investigated the neuronal expression of Beclin 1 and Caspase-3 as characteristic markers in the autophagic promotion of post-TBI apoptosis.

Treatment with co-ultra PEALut significantly reduced the number of Beclin 1/Caspase-3 positive hippocampal neurons at 72 h after injury, and even more 7 d after TBI.

The hallmark of CNS trauma is the increase in reactive astrocytes translated into elevated GFAP expression [60]. The role of elevated GFAP immunoreactivity in CNS injury is quite complex because the astrocytic response can reflect reparative and/or pathological processes depending on the period elapsed post-injury [61]. Reactive gliosis is essential for the protective role of astrocytes in the acute stages of neurotrauma. However, GFAP is also linked to neural plasticity and regenerative responses in the healthy and injured brain [62]. Accordingly, it has been shown that GFAP mice deficient (GFAP −/−) showed greater post-traumatic synaptic plasticity as well as basal and post-traumatic hippocampal neurogenesis [62].

Furthermore, astrocytes respond to pro-inflammatory stimuli from peripheral immune cells such as mast cells [63]. This crosstalk plays a key role in the neuroinflammatory processes underlying many brain disorders [63].

Our data revealed an apparent increase in GFAP positive cells in the DG as well as in the cortex following TBI; astrogliosis was notably reduced after treatment with co-ultra PEALut. We speculate that considering the crosstalk between mast cells and astrogliosis, treatment with co-ultra PEALut could decrease gliosis through mast cell modulation by Autacoid Local Injury Antagonism (ALIA) mechanism [64].

Another typical sign of CNS damage is the increase in reactive microglia, revealed by the overexpression of Iba1 [65].

In this regard, Sun et al. [66] highlighted how neuroinflammation is linked with microglial activation in the hippocampus after TBI. This condition consequently leads to hippocampal neuroinflammation, which can disrupt cognitive function directly or indirectly through decreasing neurogenesis [66].

In agreement with this, our results showed a significant increase in Iba1 expression in the hippocampus and the cortical impact area at both 72 h and 7 d post-TBI.

Conversely, treatment with co-ultra PEALut was able to considerably reduce the upregulation of Iba1 induced by brain trauma.

Together, these results demonstrated the ability of co-ultra PEALut association to suppress the neuroinflammation seen in TBI by decreasing astrocyte activation and reactive gliosis in the hippocampus and cortex, thus improving neurogenesis processes. 

It has been shown that neurotrophins regulate many factors of neuronal survival including dendrite reduction, axon growth, synaptic function, and plasticity [67]. 

Moreover, it is well known that neurotrophins such as BDNF or NT-3 promote the regeneration of damaged brain tissues [20]. Our study clearly showed that BDNF and NT-3 levels were downregulated after TBI compared to sham mice. 

Interestingly, treatment with co-ultra PEALut considerably enhanced neurotrophic factors. On this basis, we supposed that co-ultra PEALut could improve neurogenesis exerting neurogenic and neuroprotective through the stimulation of neurotrophic production. Blázquez et al. argues the relevance of the CB1/BDNF axis in promoting neuron survival [68]. PEA could act through these cannabinoid receptors. On the other hand, it has been shown that Lut upregulates the synthesis and release of neurotrophins; this capacity appears to be mediated through estrogen signaling pathways [69]. However, further analyses are needed to better understand the mechanisms underlying the regulation of neurotrophins by co-ultra PEALut.

In the clinical study, it was indicated, for the first time, that co-ultra PEALut (Glialia^®^) may help to facilitate neural recovery on working memory and executive functioning following moderate TBI, with small to moderate effect sizes. No side effects were stated by participants, supporting the safety and tolerability profile of Glialia^®^ post-TBI, according to other clinical studies [70].

Memory impairment represents one of the maximum residual deficits following TBI and is among the greatest frequent complaints heard from patients and their relatives [71,72]. After emerging from the coma, patients usually cross a phase of global cognitive disorder commonly termed “post-traumatic amnesia”. At this phase, patients are disoriented in place and time and incapable of storing or recovering new info; some grade of retrograde amnesia is often present as well. Retrieval is habitually gradual, and a consistent return of permanent memory denotes post-traumatic amnesia (PTA) clearing. However, compromised memory frequently persists later. This favorable effect of Glialia^®^ treatment may be related to the demonstrated in vivo data showing a reduction in apoptotic cell death, inflammation, edema, and improving tissue structure, as well as reduction of microglial cell and astrocyte reactivity in the lesioned area [35,73]. On the other hand, our results should be considered preliminary and taken with caution because the study power was limited due to the low number of patients recruited because of restrictive inclusion and exclusion criteria and length of Glialia^®^ administration.

Thus, larger, multicenter trials are necessary to provide definitive evidence.

In summary, the search for molecules that contribute to the neuro-regenerative process becomes important in view of a potential implication to protect the permanent loss of neurons after head trauma. Certainly, future analyses are needed to study in depth the mechanism of action of this compound and confirm these observations in a wider patient population. However, our data clearly showed that co-ultra PEALut could represent a potential therapeutic compound for the improvement of the endogenous reparation response for treating TBI—improving, in a translational way, the positive outcomes in traumatic injuries.

## 4. Materials and Methods

### 4.1. Materials

The co-ultra PEALut (commercial name: Glialia^®^) was a generous gift from Epitech Group SpA; all the other chemicals were of the highest commercial grade available. All the stock solutions were made in either nonpyrogenic saline (0.9% NaCl; Baxter, Milan, Italy) or 10% dimethyl sulfoxide.

### 4.2. Co-Ultramicronization Process for PEA and Lut

The co-ultramicronization procedure was executed using jet mill equipment (Sturtevant Inc., 348 Circuit Street Hanover, MA, USA) with a chamber of 300 mm in diameter, which was operated with “spiral technology” and driven by compressed air at 10 to 12 bars as previously described [35]. The co-ultra PEALut (Epitech Group SpA) was dissolved in 1.5% carboxymethyl cellulose (CMC) and used at concentrations of 1 mg/kg. The co-ultra PEALut 1 mg/kg was administered to mice, orally, 1 h after craniotomy, and daily for 72 h and 7 d.

### 4.3. Pre-Clinical Methods

#### 4.3.1. Animals

Male mice CD1 (25 to 30g, Envigo, Desio, MB, Italy), aged between 10 and 12 weeks, were used for the entire study. Mice were located in stainless steel cages in a room kept at 22 ± 1 °C with a 12-h dark, 12-h light cycle. The animals were familiarized with their habitat for one week and had access to rodent standard diet and water ad libitum. University of Messina Review Board for the care of animals approved the study. Animal care was in accordance with the novel legislation for the protection of animals used for scientific purposes (D. Lgs 2014/26 and EU Directive 2010/63).

#### 4.3.2. Controlled Cortical Impact (CCI) Experimental TBI

TBI was induced in animals by a controlled cortical impactor (CCI) by using the controlled impactor device Impact OneTM Stereotaxic impactor for CCI (Leica, Milan, Italy) as previously described and reported briefly below [74,75]. The craniotomy of the right hemisphere including the bregma and lambda between the sagittal suture and the coronal ridge was executed with a micro-motor handpiece and drill (UGO Basile SRL, Comerio Varese, Italy). Subsequently, once taken out the resultant bone flap, the cranial space was expanded with cranial rongeurs (New Adalat Garh, Roras Road, Pakistan). A cortical contusion was performed through the controlled stereotaxic impactor (Leica, Milan, Italy) on the uncovered cortex (tip diameter: 4 mm; cortical contusion depth: 3 mm; impact velocity: 1.5 m/s). This produced brain injury of moderate severity. Closely after injury, the skin cut was sutured with surgical staples by nylon thread and was applied 2% lidocaine jelly in the lesion in order to decrease pain. All groups were sacrificed 72 h or 7 d post-TBI-injury for histopathological and biochemical examinations.

#### 4.3.3. Experimental Groups

Mice were randomly distributed into the following groups:

1. Sham group: animals underwent identical surgical procedures except for TBI shock and were maintained under anesthesia for the period of the experiment (n = 10).
2. Sham + Co-ultra PEALut group: mice underwent indistinguishable surgical procedures except for TBI shock plus Co-ultra PEALut (1 mg/kg in 1.5% CMC, orally by gavage) administration 1 h after craniotomy and quotidian for 72 h and 7 d (n = 10). (Data not shown)
3. TBI + vehicle group: mice underwent CCI (n = 10) plus vehicle, used for co-ultra PEALut, administrated 1 h after craniotomy and quotidian for 72 h and 7 d (n = 10).
4. TBI + Co-ultra PEALut group: the mice were subjected to TBI plus administration Co-ultra PEALut (1 mg/kg in 1.5% CMC, orally by gavage) 1 h after craniotomy and quotidian for 72 h and 7 d (n = 10).

The dose and the animal model were based on previous in vivo studies [70,76,77].

#### 4.3.4. Histological Analysis

Sagittal sections of 7-μm thickness were processed from the perilesional brain region of each mouse and were valued by an experienced histopathologist. Slices were stained with hematoxylin and eosin. Histopathologic variations of the grey matter were scored on a six-point scale as previously described [73]. All the histological studies were conducted in a blinded manner.

#### 4.3.5. Immunohistochemistry (IHC)

Immunohistochemical evaluation for thymidine analog bromodeoxyuridine (BrdU), doublecortin (DCX), brain-derived neurotrophic factor (BDNF) and neurotrophin-3 (NT-3) was performed as previously described [78] and briefly reported below.

After deparaffinization, endogenous peroxidase was blocked with 0.3% (*v*/*v*) hydrogen peroxide in 60% (*v*/*v*) methanol for 30 min. Sections were rehydrated with 0.1% (*w*/*v*) of Triton X-100 in PBS for 20 min and subsequently incubated with 2% (*v*/*v*) of normal horse serum in PBS for 20 min, in order to minimize nonspecific absorption.

Slices were incubated overnight with mouse monoclonal anti-BrdU antibody (1:100 in PBS *v*/*v*; sc-32323; Santa Cruz Biotechnology, Dallas, TX, USA), goat polyclonal anti-DCX antibody (1:500 in PBS *v*/*v*; sc-271390; Santa Cruz Biotechnology, Dallas, TX, USA), anti-BDNF polyclonal antibody (1:500 in PBS *v*/*v*; sc-546; Santa Cruz Biotechnology, Dallas, TX, USA) and anti-NT-3 polyclonal antibody (1:500 in PBS *v*/*v*; sc-547; Santa Cruz Biotechnology, Dallas, TX, USA). At the end of the incubation with the primary antibody, the sections were abundantly washed with PBS and incubated with a secondary antibody (1:1000 Jackson Immuno Research, West Grove, PA, USA) for 1 h. The reaction was revealed by a chromogenic substrate (DAB), and counter-staining with Nuclear Fast Red. For a graphic display of the densitometric analyses, the percent of positive staining (DAB brown staining) was measured by computer-assisted color image analysis. The percentage area of immunoreactivity (determined by the number of positive pixels) was expressed as percent of total tissue area (red staining) within five random fields at a 40× magnification. For each experiment, 9 slices (3 sections per animal) were chosen for analysis. To prove the binding specificity for different antibodies, some sections were also incubated with only primary antibody or secondary antibody; moreover, we also performed negative controls in which the primary antibody was omitted. No positive staining was observed in these sections. For BrdU staining, the animals received intraperitoneal injections of BrdU (70 mg/kg) 1 h before sacrifice and twice a day up to 7 d after TBI. For BrdU and DCX sections, the numbers of the immunopositive cells for corresponding antibodies were counted in the DG. Data are representative of at least three independent experiments. Analyses were performed blindly. Images of 20× (50 µm scale bar) and 40× (20 µm scale bar) were shown.

#### 4.3.6. Behavioral Testing

All animals were subjected to an identical behavior test over 7 d at a different time point (72 h and 7 d). Testing was held in a silent (50–55 dB ambient noise), dedicated room. The tests are described below:

##### Morris Water Maze

Learning and memory function were assessed using a spatial acquisition task in a Morris water maze (MWM), as previously described [79,80]. Briefly, the apparatus consisted of a circular tank (100 cm × 50 cm), filled with water (23 ± 1 °C) and divided into four quadrants. A circular escape platform (10 cm × 24 cm) was placed in the middle of one quadrant, 1 cm below the surface of the water. Four trials of 90 s each were made a day for 5 consecutive days. The trial starts by placing each animal into the tank at one of three starting positions, facing the pool wall and allowing it to circumnavigate the pool in search of the escape platform. When the animal found the platform, it was allowed to remain on it for 20 s. the time in the platform (second) was recorded to indicate the learning results [81]. The percentage of swimming in the quadrant of the prior platform was analyzed as a measure of spatial memory; in particular, the following parameters were considered: (1) Latency to platform on day 3 with visible platform (adaptive memory and learning); (2) Latency to platform on day 7 with visible platform (adaptive memory and learning) [79].

##### Rotarod Test

The rotarod test involved analyses of motor asymmetry; the retard treadmill (Accuscan, Inc., Columbus, OH, USA) provided a motor balance and coordination assessment. Data were generated by averaging the scores (total time spent on the treadmill divided by 5 trials) for each animal during training and testing days as previously described [82]. 

#### 4.3.7. Immunofluorescence of GFAP, Iba1, NeuN, Caspase-3 and Beclin 1

Immunofluorescence was performed as previously described [20].

After deparaffinization and rehydration, as for IHC protocol, slides were placed in 0.1 M citrate buffer for one minute. Nonspecific absorption was decreased by incubating the sections in a solution containing 2% (*v*/*v*) of normal goat serum in PBS for 20 min. Then, brain tissue sections were incubated with anti-GFAP (1:100; sc-9065; Santa Cruz Biotechnology, Dallas, TX, USA), anti-Iba1 (1:100; sc-32725; Santa Cruz Biotechnology, Dallas, TX, USA), anti-NeuN (1:100; MAB377; Millipore, Burlington, MA, USA), anti-caspase-3 (1:100; sc-7272; Santa Cruz Biotechnology, Dallas, TX, USA) and anti-Beclin 1 (1:100; sc-11427; Santa Cruz Biotechnology, Dallas, TX, USA) the whole process was performed overnight in a humidified chamber at 37 °C. 

Sections were washed with PBS and incubated with Alexa Fluor-488 FITC-conjugated anti-mouse antibody (1:1000 *v*/*v* MolecularProbes, Manchester, UK), for 1 h at 37 °C.

The sections were washed and for nuclear staining 2 μg/mL of 4’,6’-diamidino-2-phenylindole (DAPI; Hoechst, Frankfurt; Germany) was added in PBS. Sections were observed and acquired at 40× magnification using a Leica DM2000 microscope (Leica, Milan, Italy). To prove the binding specificity for different antibodies, some sections were also incubated with only primary antibody or secondary antibody, no positive staining was observed in these sections. 

#### 4.3.8. FluoroJade

To assess neuronal degeneration, brain sections were stained with Fluoro-Jade C (Millipore, Burlington, MA, USA) as previously described [77]. Briefly, slides were immersed in 80% ethanol with 1% NaOH for 5 min, followed by 2 min in 70% ethanol, 2 min in distilled water, and incubated in 0.06% potassium permanganate solution for 10 min. Subsequently, sections were rinsed in water and then transferred to a 0.001% solution of Fluoro-Jade C in 0.1% acetic acid. The slides were then washed in distilled water, air-dried, cleared in xylene and coverslipped with DAPI. ImageJ was employed to quantify green Fluoro-Jade C staining.

#### 4.3.9. Western Blot Analysis

Western blot analysis was performed as described [83].

Briefly, after protein extraction from brain tissues, cytosolic lysates were used for the detection of BDNF and NT-3.

After SDS-PAGE, the proteins present in the polyacrylamide gel are transferred to the PVDF membrane. Membranes were incubated at 4 °C overnight with each of the following primary antibodies: anti-BDNF (1:500; sc-20981; Santa Cruz Biotechnology, Dallas, TX, USA), anti-NT-3 (1:500; sc-547; Santa Cruz Biotechnology, Dallas, TX, USA) dissolved in a PMT solution consisting of 1× phosphate buffer saline (PBS), 5% *w*/*v* nonfat dried milk powder and 0.1% Tween-20. Thereafter, the membranes were washed and incubated with secondary antibody (1:1000, Jackson ImmunoResearch, West Grove, PA, USA) for 1 h at room temperature.

To confirm that the samples used contained a uniform concentration of protein lysates, they were incubated in the same way, with primary anti-β-actin antibody (1:500; sc-47778; Santa Cruz Biotechnology, Dallas, TX, USA). Signals were exposed with chemiluminescence (ECL) detection system reagent according to the manufacturer’s instructions (Thermo, Waltham, MA, USA). The relative expression of the protein bands was quantified by densitometry and standardized to β-actin levels, as an internal control.

#### 4.3.10. Statistical Analysis

All values are expressed as mean ± SEM of N observations. For the in vivo studies, N is the number of animals reviewed. In the trials concerning histology or immunohistochemistry, the figures represent a minimum of three experiments executed on different experimental days. The data were examined by One-way and Two-way ANOVA test followed by a Bonferroni post-hoc test for multiple comparisons. A *p*-value smaller than 0.05 was considered significant. 

### 4.4. Clinical Methods

#### 4.4.1. Study Setting

Between May 2016 and October 2017, all patients affected by moderate TBI (Glasgow Coma Score 9–13) admitted to the neurosurgical department of the University of Messina, Italy were screened for recruitment. 

#### 4.4.2. Sample Size

In order to spot a possible superiority of the experimental treatment over the standard therapy, a sample size analysis was performed. In order to evaluate the effects on the cognition and memory of patients affected by moderate TBI, we selected the mini mental state examination (MMSE) to set the sample size. According to literature data, the MMSE score after moderate TBI is 24.2 (4.9 SD). Considering an improvement of 10% (i.e., MMSE score of 24) and an α of 0.05 and a power of 80%, we calculated that 60 patients must be included in the study. Furthermore, considering a possible 10% dropout, we considered the necessity to recruit 66 patients.

#### 4.4.3. Randomization

Patients of the sample were randomized with a 1:1 ratio and included in the two branches of the study (Standard Treatment vs. Standard Treatment + Glialia^®^) using a precompiled list of selection. 

#### 4.4.4. Primary Endpoint 

The primary endpoint of the study was the effect of the experimental treatment on the functional and neurological recovery in patients with moderate TBI at a follow-up of 6 months. For this purpose, we used different evaluation scales:

(i) TBI severity assessment by the Glasgow Coma score and the Marshal Score;
(ii) Impairment of cognitive abilities by the Mini Mental State Examination (MMSE) [84] adjusted for educational level and age and the Brief Neuropsychological Cognitive Examination (BNCE) [85];
(iii) Depression by the Beck inventory depression scale;
(iv) Independence in activities of daily living and self-care by the Barthel Index.

#### 4.4.5. Blinding

The neurological and neuropsychological examination was performed initially before randomization. After inclusion in the study, patients were examined by a blinded neuropsychologist.

#### 4.4.6. Secondary Endpoints

We reported any possible adverse effects of Glialia^®^ as well as the effects on blood pressure and metabolism, which could potentially affect patient care. To this purpose, during rehabilitation therapy, patients underwent regular hematology tests and were monitored for episodes of adverse events.

To evaluate any effect of Glialia^®^ on specific subscores of the evaluation scale administered during the follow-up or any neuropsychological unexpected effect reported by the patients or caregivers.

#### 4.4.7. General Treatment

All patients underwent standard treatments according to patterns of severity and in the light of distinct variables, mainly represented by the conditions of cerebral hemodynamics. These procedures can be summarized as follows:

- Surgical evacuation of hemorrhagic masses and/or “debridement” of outbreaks brain contusion;
- Medical management aimed at the maintenance of euvolemia and adequate brain perfusion. Goals were a systolic blood pressure >90 mmHg and a cerebral perfusion pressure >60 mmHg. Prevention of secondary complications of critical illness included preventive therapy of venous thromboembolism (VTE) using low molecular weight heparin (LMWH) and seizures using levetiracetam prophylactically for the first seven days after injury;
- Patients who underwent surgical treatment also received post-operative intensive care treatments including position of the head high, lower values of end-tidal CO_2_, sedation with reduced metabolic consumption of O_2_, increase in plasma osmolarity by administration of mannitol in controlled doses or hypernatremia, therapeutic CSF drainage.

#### 4.4.8. Specific Treatment

The investigations agent, (Glialia^®^ 700 mg + 70 mg), have been administered orally twice daily (every 12 h) for 180 d in association with the specific therapy (e.g., antiplatelet agents, anticoagulants, antiepileptic drugs) commonly administered to these patients and/or with drugs prescribed for comorbidities (i.e., diabetes, arterial hypertension). The treatment with Glialia^®^ was started as soon as possible in relation to the patient’s neurological conditions (ability to effectively take oral therapy) and the inclusion in the experimental study that usually is in a period between 24 and 72 h after the traumatic event.

#### 4.4.9. Statistical Analysis

Statistical analysis was accomplished using the Student’s T test and the Mann–Whitney U test for continuous variables and X^2^ test for categorical data. The data analysis was executed by Prism, version 4.0 (GraphPad, San Diego, CA, USA). The values are expressed as the mean ± standard deviation (SD). Only a *p*-value smaller than 0.05 was considered significant.

## 5. Patents

Salvatore Cuzzocrea is co-inventor on patent *WO2013*/*121449 A8 (Epitech Group SpA)*. Methods for the modulation of amidases capable of hydrolysing N-acylethanolamines useable in the therapy of inflammatory diseases. Moreover, Dr Cuzzocrea is also a co-inventor with Epitech group on the following patents:

1. EP 2814489
2. EP 2821083
3. EP 2985037
4. 102015000067344

## 6. Study Highlights

1. The search for molecules that contribute in the neuro-regenerative process has become important, particularly in view of the potential implication to protect the permanent loss of neuron after a head trauma.
2. The aim was to appraise the neuro-regenerative properties of a micronized compound comprised of palmitoylethanolamide (PEA) and the vegetable flavonoid luteolin (Lut), which is known as co-ultramicronized PEALut (co-ultra PEALut).
3. The co-ultra PEALut treatment helped the neuronal reconstitution process to restore the basal level of both novel neurons and mature neurons in mice
4. Co-ultra PEALut could represents a potential therapeutic compound for the improvement of the endogenous reparation response for treating TBI improving the clinical result in traumatic injuries.

## Figures and Tables

**Table 1:** Cut-off values of the Brief Neuropsychological Cognitive Examination (BNCE) results were adjusted for educational level and age.

	Age							
**Educational Level**	**15–20**	**21–30**	**31–40**	**41–50**	**51–60**	**61–70**	**71–80**	**>80**
**Low**	79	72	73	65	63	59	52	44
**High**	74	82	77	77	73	61	59	45

**Table 2:** Summary of demographic and clinical characteristics of patients at hospital admission.

	Study Group (Glialia)	Control Group	P
**N. of Patients**	15	15	NS
**Gender**	13M/2F	11M/4F	NS
**Age**	49.7 ± 17.9	53.8 ± 17.5	NS
**GCS at admission**	11.2 ± 1.3	11.2 ± 1.4	NS
**MMSE score**	21.1 ± 4.6	21.5 ± 3.1	NS
**BNCE**	52.3 ± 17.5%	47.1 ± 19.3%	NS
**Marshall Scale** I II III IV V VI	- 6 3 4 1 1	- 6 4 3 2 -	NS
**Surgical Treatment**	4	3	NS

**Table 3:** Cognitive and Independency Outcome for 180 d after TBI.

OUTCOME	Study Group	Control Group	P
MMSE score	28.1 ± 2.4	26.1 ± 2.8	0.04
Δ MMSE	7.4 ± 1.7	4.6 ± 3.9	0.02
MMSE score > 26	15/15	9/15	0.02
BNCE	73.4 ± 11.2%	63 ± 24.9%	0.13
BCNE Interference Memory	85.7 ± 25.2%	51.9 ± 30.2%	0.03
Barthel Index	98.7 ± 3.6	97 ± 5.9	0.27
Becks Depression Inventory	5.2 ± 3.7	9.9 ± 6.7	0.11

## Data Availability

The authors declare that all data and materials supporting the findings of this study are available within the article. The data that support the findings of this study are available from the corresponding author upon reasonable request.

## Abbreviations

BDNF	brain-derived neurotrophic factor
BNCE	brief neuropsychological cognitive examination
BrdU	bromodeoxyuridine
CNS	central nervous system
CT	computed tomography
CCI	controlled cortical impact
DG	dentate gyrus
DCX	doublecortin
ED	emergency department
GCS	Glasgow coma scale
IRB	institutional review board
ICU	intensive care unit
ICP	intracranial pressure
Lut	luteolin
MMSE	mini mental state examination
MWM	Morris water maze
NPCs	neural precursor cells
NSCs	neuronal stem cells
NT-3	neurotrophin-3
NS	not significant
PEA	palmitoylethanolamide
SVZ	nonventricular zone
SGZ	nongranular zone
TBI	traumatic brain injury
VTE	venous thromboembolism
